# Awareness, attitudes, and perceptions of drive-thru community pharmacy services among the general public in Malaysia during COVID-19: A cross-sectional study

**DOI:** 10.1371/journal.pone.0282991

**Published:** 2023-03-10

**Authors:** Bayan F. Ababneh, Siew Chin Ong, Rabia Hussain

**Affiliations:** Discipline of Social and Administrative Pharmacy, School of Pharmaceutical Sciences, Universiti Sains Malaysia, Seberang Perai, Penang, Malaysia; Al-Jouf University College of Pharmacy, SAUDI ARABIA

## Abstract

**Introduction:**

There is a lack of attention to drive-thru services in the community pharmacy setting, particularly during the COVID-19 period in Malaysia. The main objective of this study was to assess the public awareness, attitudes, and perceptions towards drive-thru community pharmacy services among during COVID-19 in Malaysia.

**Methods:**

A cross-sectional study was conducted using a self-administrated, web-based survey (Google form) among the public in Malaysia between May and June 2022. Descriptive statistics were used to summarize the socio-demographic characteristics of the participants. Associations between the socio-demographic characteristics of the participants and the use of drive-thru community pharmacy services were assessed using a chi-square test. Regression analyses were carried out to determine whether the socio-demographic characteristics of the participants were associated with perceptions towards drive-thru community pharmacy services.

**Results:**

A total of 565 (70.6%) of the general public completed the survey instrument. The median age of study participants was 40.0 (IQR = 36.0) and about half of them were males (50.6%, n = 286). Although 18.6% (n = 105) of the participants reported the presence of DTCPS in their cities, only 9.0% (n = 51) reported having used this service. Most of the participants were supportive to establish drive-thru services at community pharmacies in the country. Most of the believed advantages among participants were that DTCPS are helpful during COVID-19 and quarantine time 48.0% (n = 271) by enhancing social distancing and reducing the spread of the COVID-19 virus 48.5% (n = 274). Among sociodemographic factors, non-Malaysian nationality (p<0.001), and age above 55 years (p = 0.01) were found to negatively affect participants’ perceptions towards drive-thru community pharmacy services.

**Conclusion:**

This study showed positive awareness, attitudes, and perceptions toward drive-thru community pharmacy services during COVID-19 in Malaysia among the public. The participants believed that those services were helpful during COVID-19 to enhance social distancing and to reduce the spread of the COVID-19 virus.

## Introduction

The World Health Organization (WHO) declared that Coronavirus disease (COVID-19) constituted a Public Health Emergency of International Concern (PHEIC) on January 30, 2020 [1]. In response to the widespread of COVID-19, WHO has declared safety precautions to prevent the spread of the virus as much as possible. One of those precautions was social distancing by maintaining at least a meter distance between one person and another [1]. Online shopping and drive-thru services became essential ways to fulfill peoples’ demands safely during lockdowns and border restrictions [2,3]. The drive-thru model was a good choice that provided a social distancing strategy [4].

Drive-thru is a way of receiving services or products without leaving the vehicle [5]. The drive-thru pharmacy service was introduced in different countries such as the United States of America, the United Kingdom, Croatia, Malaysia, Jordan, Taiwan, Qatar, and others [6,7]. It was introduced to reduce waiting time in pharmacies, to improve the availability and provision of healthcare services for the targeted population, and recently to improve safety during COVID-19 and others [7].

In the 1990s, to improve the provision of healthcare services for less mobile senior citizens, the first drive-thru pharmacy service was initiated by Walgreens pharmacy, in the United States of America (USA) [8]. Likewise, in the United Kingdom (UK), the first drive-thru pharmacy was introduced to serve elderly citizens and busy customers [9]. In Croatia, parents with young children were targeted to be served in addition to less mobile senior citizens by establishing the first drive-thru pharmacy services [10].

Drive-thru pharmacy is one of the services that was provided by the Malaysian Pharmaceutical Services Division, Ministry of Health Malaysia in 2003 as part of the Pharmacy Value Added Service (VAS) [11]. For example, the first drive-thru pharmacy in Malaysia was established in 2008 in Pulau Pinang as part of an experimental project [12]. Then, it was reported in 2014 by the Pharmaceutical Services Division, Ministry of Health Malaysia that the drive-through pharmacy service was introduced at 18 hospitals and 18 health clinics to reduce parking issues and resolve congestion in the pharmacy waiting area [13].

Furthermore, the Queen Elizabeth Hospital (QEH), Kota Kinabalu offered the drive-thru pharmacy service in 2015. Siung Liew et al. demonstrated that patients who used the drive-thru pharmacy service at (QEH) were satisfied with the service [14]. Another study in Malaysia was conducted in Hospital Raja Perempuan Zainab II (HPRZ II), in which patients were aware about the presence of drive-thru pharmacy service and assured of the importance of using this service by the public [15].

Drive-thru pharmacy Griffith has been providing time-saving pharmacy services in Australia to the community since 2010 [16]. Similarly, in Jordan, positive feedback towards using drive-thru community pharmacy services (DTCPS) was reported by pharmacy customers, especially busy customers who are males, married, and have children as they confirmed that such services are fast and time-saving [17]. Moreover, in Taiwan, the first drive-thru pharmacy was found to offer fast and efficient services compared to traditional pharmacy services [18]. However, drive-thru pharmacies may reduce interactions between the pharmacist and patient which greatly affects the counseling process. This evidence is further supported by the assessment of awareness, perception, and barriers among pharmacists in Jordan. On the other hand, drive-thru pharmacies can provide convenient dispensing of medications and solve the limited parking slots problem; thereby improving patient satisfaction [19].

During COVID-19 some countries-initiated drive-thru pharmacies for the first time to ensure the safety for pharmacists and consumers [20]. For example, in Qatar, the first drive-thru pharmacy had been introduced by Sidra Medicine for dispensing medications at the outpatient building [21]. Additionally, in the United Arab Emirates (UAE) to reduce the spread of COVID-19 infection and promote social distancing, the drive-thru pharmacy service was initiated by Thumbay University hospital [22]. The head of the Malaysian Pharmacy Association stated that the drive-thru pharmacy service is the best way that needed to be available during the COVID-19 pandemic [23].

The COVID-19 pandemic led to some economic benefits for pharmaceutical services, in which more sales and production of medications were needed; and fast-serving pharmaceutical services such as drive-thru service were highly desired by consumers [6]. The gained experiences from using drive-thru pharmacies during COVID-19 in different countries may promote the benefits that could be gained from establishing these services by standardizing global guidelines for drive-thru pharmacies [7]. A cross-sectional study was conducted in Saudi Arabia to evaluate the need for drive-thru pharmacy services as an impact of COVID-19, the result supported that there was a crucial need to support the community with drive-thru pharmacy services. However, this result is limited because the survey was only conducted in Saudi Arabia [24].

There is a lack of attention to drive-thru services in the community pharmacy setting, particularly during the COVID-19 period in Malaysia, since the previously conducted studies focused on the drive-thru pharmacy in government hospitals and before the COVID-19 period [14,15], and drive-thru community pharmacy service is relatively new [23].

The first community pharmacy that provides drive-thru services was initiated by Superbig Kubang Kerian Pharmacy on 5th February 2022, as a need during the COVID-19 pandemic since customers are more likely to visit community pharmacies to get vitamins, hygiene products, masks, and even their regular medications to avoid going to the hospitals and clinics and to enhance social distancing [23]. This community drive-thru pharmacy could decrease pressure on hospital pharmacies [23]. Hence, this study aimed to explore the awareness, perceptions and believed advantages and disadvantages of drive-thru community pharmacies among the general population in Malaysia during COVID-19. Additionally, socio-demographic factors affecting the perceptions and use of drive-thru community pharmacy services were explored.

## Materials and methods

### Study design and setting

A descriptive, cross-sectional study was conducted in Malaysia between 19th May 2022 until 22nd June 2022. A non-probability convenience sampling method was used for data collection. A self-administered instrument using an online Google form was utilized as a data-collecting tool and was optimized as well to be easily filled using computers and smartphones. Was then distributed to the participants by research assistants (undergraduate pharmacy students at Universiti Sains Malaysia) via social media platforms such as WhatsApp, Instagram, and Telegram applications. The time taken to complete answering the instrument was approximately 5 to 7 minutes. Study approval was obtained from the Human Research Ethics Committee of Universiti Sains Malaysia (USM) (Reference code: USM/JEPeM/21110755). Moreover, the participants who agreed to take part in the study signed the consent form electronically before proceeding to the first section of the instrument.

### Participants

The sample size was determined using the following formula [25] which revealed that the sample size needed to be at least 363 participants, $n=z2\times \hat{\rho }(1-\hat{\rho })÷\varepsilon 2,z$ is the z score, *ε* is the margin of error, *n* is the population size, and $\hat{\rho }$ is the population proportion. Z for a confidence level of 95% is 1.96. The margin of error is 5%. Assume a population proportion of 0.6 as one study in Malaysia revealed 60% awareness regarding the drive-thru service [15], and unlimited population size. In case of missing data, a higher sample size was recruited (565). Participants were recruited throughout Malaysia. All citizens who currently reside in Peninsular Malaysia, Sabah, and Sarawak as well as three federal territories which comprise Kuala Lumpur, Labuan, and Putrajaya were invited to participate. Inclusion criteria included those who are 18 years old and above and reside in Malaysia, have access to the internet via computer or smartphone to answer the instrument through online platforms and read and understand English.

### Study instrument

A self-administered web-based survey (Google form) instrument was used for data collection. The instrument has been designed, validated, and presented in English based on an extensive literature review [14,15,17,19,24], and some suggested questions by community pharmacists working in Malaysia. To ensure the comprehensibility and content validity of the instrument formulated, experienced lecturers at Universiti Sains Malaysia (USM) were invited to review and go through the content of the questionnaire before distributing it to the participants. Any amendments were made based on the feedback and suggestion received. After that, a pilot test was conducted among 36 participants (around 40 instruments were distributed for the pilot test, a response rate of 90.0%) who were selected conveniently from the general public and excluded from the final analysis to test the instrument before being distributed to the participants. The pilot test was conducted to identify and resolve the potential problems, and deficiencies in the instrument [26]. To ensure the reliability of the instrument, internal consistency which measures how closely related the items are for each scale was calculated using Cronbach’s alpha coefficient for perceptions score, with values of 0.70 and above indicating good internal consistency [27].

The instrument design was divided into three main sections with forty-nine items. This included participants’ demographic information, attitudes toward DTCPS, and perceptions towards DTCPS. The objectives of this research study had been covered in these three sections. Items for the first section and second section which are socio-demographic information and attitudes of the general public towards DTCPS respectively were designed in open-ended questions and multiple-choice questions. Items for the third section discussed the perceptions, believed advantages, and believed disadvantages towards DTCPS, were designed in Likert Scale-type questions (5 = strongly agree, 4 = agree, 3 = neutral, 2 = disagree, and 1 = strongly disagree), and a reverse coding considered for negative statements and for the disadvantages (1 = strongly agree, 2 = agree, 3 = neutral, 4 = disagree, and 5 = strongly disagree), to indicate the degree of agreement or disagreement of participants after they went through the respective statements in this section. A total score of perceptions out of 140 was calculated. The higher the mean perceptions score the more positive the participants perceptions towards DTCPS.

### Statistical analysis

Descriptive statistics were used to summarize the socio-demographic characteristics of participants. Categorical variables data were presented as frequency and percentage. The mean, standard deviation (SD), median, and interquartile range were reported for continuous data. To assess the normality, Kolmogorov–Smirnov test was performed. Since the data did not support parametric assumptions, the Kruskal-Wallis test and Mann-Whitney U test were performed when applicable. For multiple group comparisons, Bonferroni correction was applied, and the results were evaluated accordingly. To assess the association between categorical variables chi-square test was performed. Multiple linear regression was carried out to determine whether the socio-demographics of participants (independent variables of the analysis) were associated with the perceptions of participants (which are the dependent variables of the analysis). Variables significant at a p-value of less than 0.10 were included in the multiple linear regression analysis. Preliminary analyses ensured that there were no violations of assumptions of normality and multi-collinearity before fitting the multiple regression model (VIF for each variable was < 10). ‏ The internal consistency of the instrument scales was assessed using Cronbach’s alpha. A p-value of 0.05 was used for all statistical tests, all statistical tests were two-tailed. Analyses were performed using IBM statistical package for social science (IBM SPSS) version 28 (SPSS Inc., Chicago, IL, USA).

## Results

### Socio-demographic characteristics of participants

**Fig 1:**
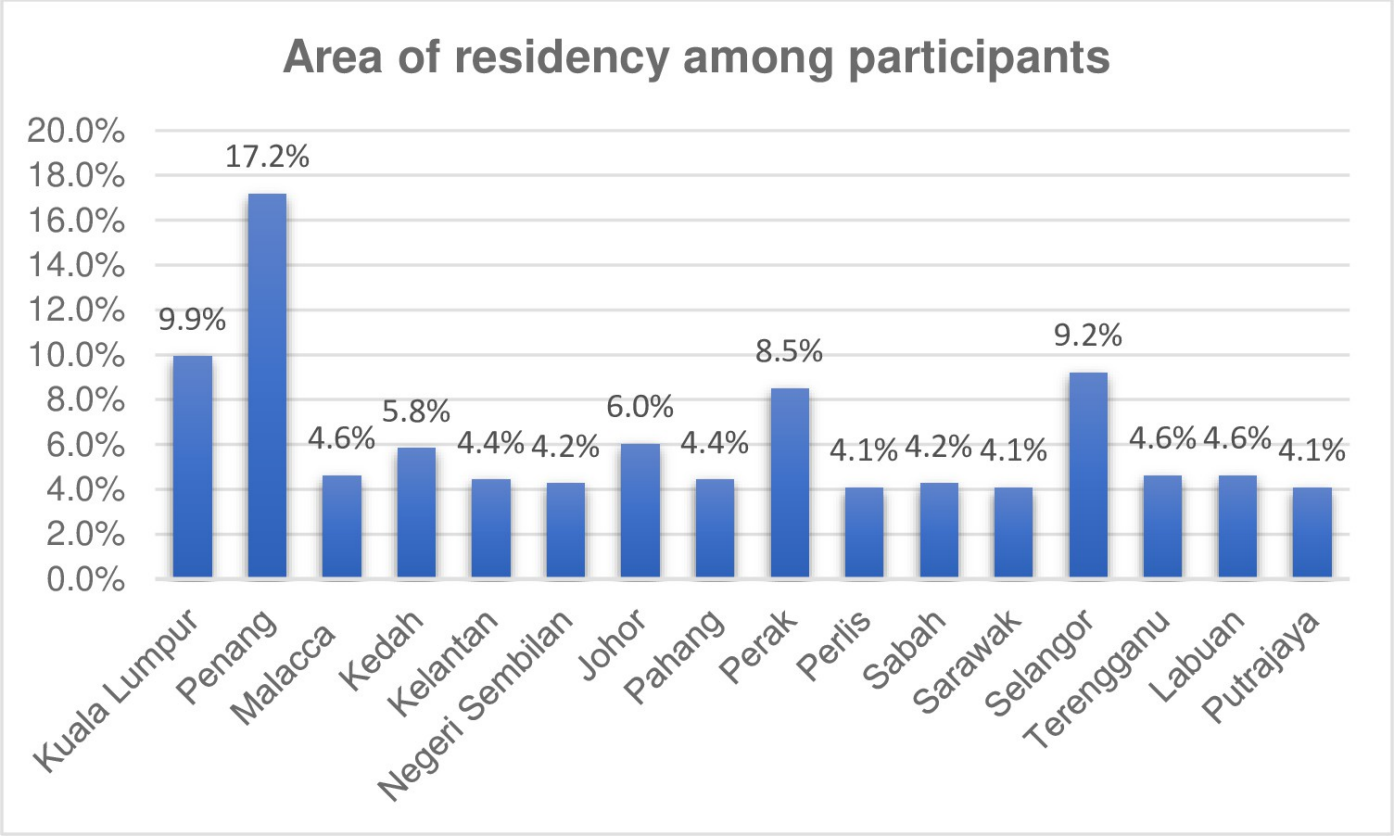
Area of residency among participants (N = 565).

Eight hundred instruments were distributed to the public in Malaysia. A total of 565 (70.6%) of the general public completed the survey instrument. The median age of the participants was 40, with an interquartile range (IQR) of 36, and about half of them were males (50.6%). Briefly, the majority were single (46.9%), have no children (51.9%), Malay (94.2%), from Penang (17.2%), had a bachelor’s degree (49.4%), and were non-employed (42.8%). Table 1 and Fig 1 summarize the socio-demographic data of all participants.

**Table 1:** General population: Socio-demographics (N = 565).

Variables	n (%)
**Age (Years), Median (IQR)**	40.0 (36.0)
18–25	204 (36.1%)
26–35	66 (11.7%)
36–45	37 (6.5%)
46–55	94 (16.6%)
Above 55	164 (29.0%)
**Nationality**	
Malaysian	532 (94.2%)
Non- Malaysian	33 (5.8%)
**Gender**	
Female	279 (49.4%)
Male	286 (50.6%)
**Marital Status**	
Single	265 (46.9%)
Married	244 (43.2%)
Divorced	23 (4.1%)
Widowed	33 (5.8%)
**Having Children**	
Yes	272 (48.1%)
No	293 (51.9%)
**Education Level**	
No formal education	20 (3.5%)
Primary school	43 (7.6%)
High school	87 (15.4%)
Diploma	58 (10.3%)
Pre-University	32 (5.7%)
Bachelor’s degree	279 (49.4%)
Master’s or Ph.D. degree	46 (8.1%)
**Employment**	
Employed	197 (34.9%)
Non-employed	242 (42.8%)
Retired	126 (22.3%)
**Health professional**	
Yes	98 (17.3%)
No	467 (82.7%)
**Student**	
Yes	215 (38.1%)
No	350 (61.9%)

IQR: Interquartile range.

### Awareness of participants about the drive-thru community pharmacy services during COVID-19

Less than half of the participants revealed that they don’t know about the presence of DTCPS at their city 44.8% (n = 253), only 9.0% (n = 51) of them used this service previously, and 6.7% (n = 38) evaluated their experience with using the service as a good experience. Most of the participants visited only one community pharmacy during the last month (44.1%, n = 249). Buying COVID-19 prevention supplies such as masks and hygiene products was the most common reason for the participants to visit the community pharmacies (60.0%, n = 339), followed by buying over-the-counter (OTC) medications (55.4%, n = 313). Most participants reported that they did not know any information about DTCPS (34.7%, n = 196). On the other hand, the internet and friends or colleagues were the main sources that triggered their awareness about the presence of the DTCPS (33.1%, n = 187), (32.9%, n = 186) respectively. Pharmacy staff also played their roles in spreading information concerning drive-thru community pharmacy towards the participants contributing to the (24.8%, n = 140). Table 2 summarizes the awareness of participants.

**Table 2:** Attitudes towards drive-thru community pharmacy services (N = 565).

Variables	n (%)
**Number of community pharmacies visited last month**	
None	114 (20.2%)
One pharmacy	249 (44.1%)
Two pharmacies	161 (28.5%)
Three or more pharmacies	41 (7.3%)
**Reasons to visit the community pharmacy** *	
Over-the-counter medications (OTC)	313 (55.4%)
For beauty products	127 (22.5%)
Prescribed medications	168 (29.7%)
Medical device	113 (20.0%)
Medical consultation	112 (19.8%)
Kid supply	41 (7.3%)
COVID-19 prevention supplies such as masks and hygiene products	339 (60.0%)
Others	22 (3.9%)
**Which category will benefit the most from drive-thru community pharmacy service?**	
All population	428 (75.8%)
Women	13 (2.3%)
Geriatrics	46 (8.1%)
People with disabilities	78 (13.8%)
**Presence of a drive-thru community pharmacy at your city**	
Yes	105 (18.6%)
No	207 (36.6%)
Don’t know	253 (44.8%)
**If yes, have you tried drive-thru community pharmacy services?**	
Yes	51 (9.0%)
No	181 (32.0%)
Not applicable	333 (58.9%)
**If yes, how do you evaluate your experience with drive-thru community pharmacy services?**	
Excellent	31 (5.5%)
Good	38 (6.7%)
Fair	11 (1.9%)
Poor	1 (0.2%)
Not applicable	484 (85.7%)
**If you are going to request an order at a community pharmacy using drive-thru service, what is your preferred method to do that order?**	
Through a drive-thru window	185 (32.7%)
Through WhatsApp	146 (25.8%)
Over the phone	93 (16.5%)
Online through application	124 (21.9%)
Through email	17 (3.0%)
**If you are going to use a drive-thru service at a community pharmacy, what is your preferred method to get information about your medications(counselling)?** *	
Briefly through the drive-thru window	283 (50.1%)
Printed brochure given with the order	189 (33.5%)
Written on WhatsApp	253 (44.8%)
Verbally over the phone	233 (41.2%)
Through a personal visit	168 (29.7%)
Through email	97 (17.2%)
**Where did you get information regarding drive-thru community pharmacy** *	
Pharmacy staff	140 (24.8%)
Doctors	81 (14.3%)
Leaflets	58 (10.3%)
Television	85 (15.0%)
Internet	186 (32.9%)
Friends or Colleagues	187 (33.1%)
Don’t know	196 (34.7%)
**Are you supportive to establish drive-thru services at community pharmacies?**	
Yes	548 (97.0%)
No	17 (3.0%)

*: Respondents could pick more than one answer (percentage summation≠ 100%).

### Participants attitudes towards drive-thru community pharmacy services during COVID-19

A positive attitude was found with regard to participants ‘attitudes towards DTCPS in Malaysia (Table 2). Most of the participants expressed their support to establish drive-thru service at community pharmacies in the country 97% (n = 548). More than half of the participants believed that drive-thru community pharmacy will benefit all population. A 32.7% (n = 185) of participants preferred to request an order at a community pharmacy using the drive-thru service through the drive-thru window, and about half of them 50.1% (n = 283) preferred to receive counselling while using this service briefly through the drive-thru window.

### Participants perceptions towards drive-thru community pharmacy services during COVID-19

A positive perception was found with regard to participants’ perceptions towards the DTCPS (Table 3). More than half of the participants believed that the DTCPS is a friendly service provided by the pharmacy during COVID-19 time or even later; that they support establishing drive-thru services to the community pharmacy practice during COVID-19 and all over Malaysia, and that the DTCPS may improve participants’ satisfaction with the pharmacy profession. About half of the participants believed that community pharmacists will have a good balance between the health of patients and the business side of their work.

**Table 3:** Perceptions towards drive-thru community pharmacy services (N = 565).

Variables	Strongly Disagree n (%)	Disagree n (%)	Neutral n (%)	Agree n (%)	Strongly Agree n (%)
**Perceptions towards drive-thru community pharmacy services as an impact of COVID-19 or even later on**					
I believe the introduction of drive-thru service makes the community pharmacy services more efficient.	4(0.7%)	6(1.1%)	61(10.8%)	244(43.2%	250(44.2%)
I believe that drive-thru community pharmacy service is a friendly service provided by the pharmacy during COVID-19 time or even later on.	3(0.5%)	4(0.7%)	41(7.3%)	264(46.7%)	253(44.8%)
I believe that drive-thru community pharmacy service may improve my satisfaction with the pharmacy profession.	3(0.5%)	11(1.9%)	82(14.5%)	227(40.2%)	242(42.8%)
I am supportive of the introduction of drive-thru service to community pharmacy practice during COVID-19 time.	4(0.7%)	7(1.2%)	39(6.9%)	240(42.5%)	275(48.7%)
I am supportive to create community pharmacies with drive-thru services all over Malaysia.	6(1.1%)	9(1.6%)	62(11.0%)	211(37.3%)	277(49.0%)
**How do you feel the image of the community pharmacists will be affected by the introduction of drive-thru services?**					
Community pharmacists will appear more concerned with making money than with the health of their patients. **	43(7.6%)	160(28.3%)	165(29.2%)	139(24.6%)	58(10.3%)
Community pharmacists will have a good balance between the health of patients and the business side of their work.	2(0.4%)	25(4.4%)	152(26.9%)	273(48.3%)	113(20.0%)
Community pharmacists will appear more concerned with the health of patients than with the business side of their work.	8(1.4%)	60(10.6%)	179(31.7%)	221(39.1%)	97(17.2%)
**Differences between the drive-thru community pharmacy services and in-store drug refill services**					
The prescription might be filled more quickly in drive-thru compared to in-store refill.	3(0.5%)	21(3.7%)	103(18.2%)	289(51.2)	149(26.4%)
Pharmacists might be less available to answer questions using drive-thru service compared to in-store refill. **	8(1.4%)	40(7.1%)	105(18.6%)	278(49.2%)	134(23.7%)
Written information might be less supplied using drive-thru pharmacy service compared to in-store refill. **	9(1.6%)	63(11.2%)	125(22.1%)	263(46.5%)	105(18.6%)
Pharmacists cannot explain important points about prescriptions while providing drive-thru service compared to that in-store refill. **	15(2.7%)	84(14.9%)	106(18.8%)	249(44.1%)	111(19.6%)
Drive-thru service provides accessibility and convenience to customers more than the in-store service, especially during COVID-19 time.	4(0.7%)	15(2.7%)	102(18.1%)	247(43.7%)	197(34.9%)
Unlike in-store service, drive-thru service is suitable only for refill prescriptions but not for new prescriptions. **	8(1.4%)	34(6.0%)	113(20.0%)	248(43.9%)	162(28.7%)
Unlike in-store service, drive-thru service is suitable only for OTC but not for prescriptions medications. **	11(1.9%)	49(8.7%)	150(26.5%)	230(40.7%)	125(22.1%)

**: Reversed coded statements.

### Participants-perceived differences between drive-thru and in-store drug refills during COVID-19

Table 3 shows participants -perceived differences between drive-thru and in-store drug refills. About 51.2% of the participants agreed that the prescription might be filled more quickly in the drive-thru compared to in-store pharmacy services, and 43.7% agreed that drive-thru service is more accessible and convenient compared to in-store pharmacy services, especially during COVID-19 time. However, participants agreed to the following statements pharmacist providing the drive-thru service, being less available to answer their questions (49.2%, n = 278), providing less written information (46.5%, n = 263), and cannot explain important points about prescriptions (44.1%, n = 249). Many participants believed that the drive-thru service is only suitable for refill prescriptions (43.9%, n = 248), and (40.7%, n = 230) for buying OTC products.

### Participants-perceived advantages and disadvantages towards drive-thru community pharmacy services during COVID-19

The strongly agreed advantages of the DTCPS as believed by 48.5% of participants as it enhances social distancing and reduces the spread of the COVID-19 virus, followed by being helpful during COVID-19 time and quarantine time (48.0%, n = 271). On the other hand, strongly agreed disadvantages of drive-thru services were restricting the opportunity for interaction with the pharmacist (21.1%, n = 119), followed by not being convenient services for providing drug information/counselling to patients (especially written information) (20.7%, n = 117). All other related advantages and disadvantages are presented in Table 4.

**Table 4:** Believed advantages and disadvantages towards drive-thru community pharmacy services (N = 565).

Believed advantages towards the drive-thru community pharmacy services as an impact of COVID-19	Strongly Disagree n (%)	Disagree n (%)	Neutral n (%)	Agree n (%)	Strongly Agree n (%)
Drive-thru community pharmacy service may help me get my medications on time without delay.	2(0.4%)	4(0.7%)	92(16.3%)	273(48.3%)	194(34.3%)
Drive-thru community pharmacy will be helpful during COVID-19 time and quarantine time.	1(0.2)	3(0.5%)	42(7.4%)	248(43.9%)	271(48.0%)
Drive-thru community pharmacy service has the advantage of serving sick patients, elderly, or disabled people during COVID-19 time.	1(0.2)	14(2.5%)	56(9.9%)	245(43.4%)	249(44.1%)
Drive-thru pharmacy service enhances social distancing and reduces the spread of the COVID-19 virus.	1(0.2)	5(0.9%)	52(9.2%)	233(41.2%)	274(48.5%)
Drive-thru community pharmacy service reduces the pressure on health care centers during COVID-19 time.	9(1.6%)	13(2.3%)	72(12.7%)	251(44.4%)	220(38.9%)
Drive-thru community pharmacy service is needed to be implemented in most community pharmacies during COVID-19 time or even later on for getting medications or supplies.	4(0.7%)	5(0.9%)	62(11.0%)	248(43.9%)	246(43.5%)
**Believed disadvantages towards the drive-thru community pharmacy services**	**Strongly** **Disagree** **n (%)**	**Disagree** **n (%)**	**Neutral** **n (%)**	**Agree** **n (%)**	**Strongly** **Agree** **n (%)**
Drive-thru community pharmacy service may contribute to dispensing errors due to the fast service provided. **	17(3.0%)	82(14.5%)	110(19.5%)	261(46.2%)	95(16.8%)
Drive-thru community pharmacy service may contribute to communication errors between the patient and pharmacist. **	15(2.7%)	72(12.7%)	91(16.1%)	280(49.6%)	107(18.9%)
Drive-thru community pharmacy service may need extra money to offer drive-thru windows. **	32(5.7%)	72(12.7%)	134(23.7%)	237(41.9%)	90(15.9%)
Drive-thru community pharmacy service is not convenient in providing drug information/counselling to patients (especially written information). **	16(2.8%)	92(16.3%)	109(19.3%)	231(40.9%)	117(20.7%)
Getting prescriptions dispensed as quickly as possible using drive-thru community pharmacy service, the quality of pharmacy service will drop. **	38(6.7%)	101(17.9%)	152(26.9%)	204(36.1%)	70(12.4%)
Drive-thru community pharmacy service restricts the opportunity for interaction with the pharmacist because the customer feels they can’t ask questions while they’re being hurried through. **	17(3.0%)	69(12.2%)	103(18.2%)	257(45.5%)	119(21.1%)
Drive-thru community pharmacy service restricts the opportunity for interaction with the pharmacist because the pharmacist will not be able to offer any level of interaction. **	21(3.7%)	75(13.3%)	110(19.5%)	243(43.0%)	116(20.5%)
**Total perceptions score Median (IQR)** **Cronbach’s alpha: 0.84**	**92.0 (16.0)**				

**: Reversed coded statements.

### Reliability and validity of the study instrument

Face and content validity of the study instrument were satisfactory as found by the expert panel. The Cronbach’s alpha coefficient for the total perceptions score was 0.84 (Table 4), which shows that the data exhibited strong internal consistency in the format used.

### Factors associated with participants’ use of drive-thru community pharmacy service during COVID-19

The relationship between the socio-demographic factors and participants’ total perceptions score towards DTCPS is shown in Table 5. Age above 55 years (p <0.001), male gender (p = 0.006), married marital status (p = 0.001), having children (p = 0.004), having bachelor’s degree (p = 0.001), being employed (p = 0.002), and not being from a medical team (p = 0.006) or students (p = 0.002), were found to positively affect participants’ use of the DTCPS during COVID-19.

**Table 5:** Factors associated with the use of drive-thru community pharmacy services among the general population using Univariable analysis (N = 565).

Factors	n (%)	p-value
**Age**		<0.001*
18–25	12 (5.9)	
26–35	6 (9.1)	
36–45	10 (27.0)	
46–55	7 (7.4)	
Above 55	16 (9.8)	
**Nationality**		0.727
Malaysian	47 (8.8)	
Non- Malaysian	4 (12.1)	
**Gender**		0.006*
Female	21 (7.5)	
Male	30 (10.5)	
**Marital Status**		0.001*
Single	17 (6.4)	
Married	26 (10.7)	
Divorced	5 (21.7)	
Widowed	3 (9.1)	
**Having Children**		0.004*
Yes	29 (10.7)	
No	22 (7.5)	
**Area of residency**		0.058
Kuala Lumpur	11 (19.6)	
Penang	6 (6.2)	
Malacca	1 (3.8)	
Kedah	1 (3.0)	
Kelantan	3 (12.0)	
Negeri Sembilan	3 (12.5)	
Johor	6 (17.6)	
Pahang	4 (16.0)	
Perak	0 (0)	
Perlis	2 (8.7)	
Sabah	2 (8.3)	
Sarawak	3 (13.0)	
Selangor	4 (7.7)	
Terengganu	1 (3.8)	
Labuan	1 (3.8)	
Putrajaya	3 (13.0)	
**Education Level**		0.001*
No formal education	1 (5.0)	
Primary school	0 (0)	
High school	6 (6.9)	
Diploma	7 (12.1)	
Pre-University	0 (0)	
Bachelor’s degree	31 (11.1)	
Master’s or Ph.D. degree	6 (13.0)	
**Employment**		0.002*
Employed	27 (13.7)	
Non-employed	13 (5.4)	
Retired	11 (8.7)	
**Health professionals**		0.006*
Yes	15 (15.3)	
No	36 (7.7)	
**Student**		0.002*
Yes	15 (7.0)	
No	36 (10.3)	

*: The Chi-square statistic is significant at the 0.05 level.

### Factors associated with participants’ perceptions towards drive-thru community pharmacy service during COVID-19

The relationship between the socio-demographic factors and participants’ perceptions towards DTCPS is shown in Tables 6 and 7. Non-Malaysian nationality and age above 55 years were found to negatively affect participants’ perceptions towards DTCPS (p <0.001), (p = 0.01) respectively.

**Table 6:** Association between perceptions and sociodemographic factors among the general population (N = 565).

Variable	Total perception score	p-value
	Median (IQR)	
**Age**		0.001*
18–25	93.5(16.0)	
26–35	90.0(16.0)	
36–45	91.0(17.0)	
46–55	97.0(18.0)	
Above 55	88.0(14.8)	
**Nationality**		0.0001*
Malaysian	92.5(16.0)	
Non- Malaysian	86.0(6.5)	
**Gender**		0.49
Female	92.0(16.0)	
Male	92.0(17.0)	
**Marital Status**		0.48
Single	93.0(16.0)	
Married	91.0(17.0)	
Divorced	88.0(12.0)	
Widowed	92.0(16.0)	
**Having Children**		0.27
Yes	91.0(17.0)	
No	92.0(16.0)	
**Area of residency**		0.80
Kuala Lumpur	98.5(17.8)	
Penang	90.0(16.0)	
Malacca	91.5(14.5)	
Kedah	93.0(13.5)	
Kelantan	93.0(18.0)	
Negeri Sembilan	92.5(14.8)	
Johor	92.0(14.5)	
Pahang	88.0(16.0)	
Perak	94.0(12.8)	
Perlis	86.0(17.0)	
Sabah	91.5(24.0)	
Sarawak	90.0(11.0)	
Selangor	97.0(16.5)	
Terengganu	91.0(16.0)	
Labuan	98.0(19.5)	
Putrajaya	88.0(19.0)	
**Education Level**		0.10
No formal education	88.5(15.5)	
Primary school	86.0(11.0)	
High school	94.0(16.0)	
Diploma	95.0(16.3)	
Pre-University	89.5(15.5)	
Bachelor’s degree	92.0(16.0)	
Master’s or Ph.D. degree	96.0(21.3)	
**Employment**		0.10
Employed	93.0(16.0)	
Non-employed	92.0(16.0)	
Retired	89.0(15.3)	
**Health professionals**		0.04*
Yes	92.0(20.3)	
No	92.0(15.0)	
**Student**		0.25
Yes	92.0(16.0)	
No	91.0(17.0)	

Kruskal–Wallis and Mann–Whitney U tests were applied where applicable, *: The median difference is significant at the 0.05 level.

**Table 7:** Factors associated with perceptions towards drive-thru community pharmacy services among the general population using Multiple linear regression analysis (N = 565).

Constant	Unstandardized Coefficients		Standardized Coefficients		(95% CI)	p-value
	B	Std. Error	Beta	t		
**Age**						
26–35	-2.78	1.94	-0.08	-1.43	(-6.60–1.03)	0.15
36–45	-3.63	2.43	-0.08	-1.49	(-8.41–1.15)	0.14
46–55	-0.11	1.94	-0.00	-0.05	(-3.92–3.72)	0.96
Above 55	-4.85	1.92	-0.20	-2.52	(-8.63- -1.07)	0.01*
**Nationality**						
Non-Malaysian	-8.41	2.15	-0.18	-3.91	(-12.64- -4.18)	<0.001*
**Area of residency**						
Kuala Lumpur	0.63	1.89	0.02	0.33	(-3.08–4.34)	0.74
Malacca	0.27	2.49	0.01	0.11	(-4.62–5.17)	0.91
Kedah	-1.72	2.25	-0.04	-0.76	(-6.14–2.70)	0.45
Kelantan	1.97	2.48	0.04	0.79	(-2.91–6.84)	0.43
Negeri Sembilan	2.59	2.59	0.05	1.00	(-2.49–7.68)	0.32
Johor	0.60	2.25	0.01	0.26	(-3.82–5.01)	0.79
Pahang	1.90	2.57	0.04	0.74	(-3.15–6.95)	0.46
Perak	0.22	1.99	0.01	0.11	(-3.69–4.13)	0.91
Perlis	-0.34	2.67	-0.01	-0.13	(-5.59–4.91)	0.90
Sabah	2.69	2.52	0.05	1.07	(-2.27–7.64)	0.29
Sarawak	0.77	2.66	0.01	0.29	(-4.45–5.99)	0.77
Selangor	2.68	1.91	0.07	1.40	(-1.08–6.44)	0.16
Terengganu	0.17	2.46	0.00	0.07	(-4.66–5.00)	0.94
Labuan	4.82	2.58	0.09	1.87	(-0.25–9.89)	0.06
Putrajaya	1.79	2.69	0.03	0.67	(-3.50–7.07)	0.51
**Education Level**						
No formal education	0.35	2.74	0.01	0.13	(-5.02–5.73)	0.90
Primary school	-1.91	2.05	-0.05	-0.93	(-5.95–2.12)	0.35
High school	0.16	1.62	0.01	0.10	(-3.01–3.34)	0.92
Diploma	2.46	1.69	0.07	1.45	(-0.86–5.79)	0.15
Pre-University	-0.70	2.06	-0.02	-0.34	(-4.75–3.35)	0.73
Master’s or Ph.D. degree	4.40	1.94	0.11	2.26	(0.58–8.21)	0.02
**Employment**						
Employed	0.87	1.56	0.04	0.56	(-2.19–3.93)	0.58
Retired	0.63	1.83	0.02	0.34	(-2.97–4.22)	0.73
**Health professionals**						
Yes	1.92	1.26	0.07	1.53	(-0.55–4.39)	0.13

C.I: Confidence interval.

Multiple linear regression analysis for factors associated with perceptions towards drive-thru community pharmacy services among the general population. These statistically significant variables predicted the perceptions score, F (6281.177, 29) = 1.867, p = 0.004, R2 = 9.2.

## Discussion

This study describes the Malaysian public awareness and perceptions of DTCPS during COVID-19. During COVID-19, people in many counties were obligated to get their needs including medications through online platforms such as in Brazil, China, Germany, Italy, the Republic of Korea, the Russian Federation, South Africa, Switzerland, Turkey, and the United Arab Emirates [28,29] or through drive-thru services in some countries around the world including Malaysia [6,7,23]. It is crucial to review DTCPS during COVID-19 from a public perspective to shed light on the gained experiences from using this service to improve its application as much as possible [7]. Awareness, attitudes, and perceptions of public perspectives will help in the evaluation of this service. To the best of our knowledge, this study is the first study in Malaysia that assessed awareness, attitudes, and perceptions among the general public regarding DTCPS.

Despite the high awareness among Malaysian hospital pharmacy consumers towards hospital pharmacy drive-thru services [15], it was shown in this study that about half of the participants were not aware about the presence of the DTCPS, this justifies the low rate of Malaysian public who had tried this service. This is because the drive-thru service at community pharmacy was recently introduced officially in Malaysia in February 2022 [23]. These findings were similar to previous studies conducted in Jordan and Saudi Arabia as more than half of customers were not aware of its presence in community pharmacies [17,24].

The main sources that triggered participants’ awareness in this study about the presence of DTCPS were the internet, friends or colleagues, and pharmacy staff. Surprisingly, however, only 14% of the participants in the current study reported doctors as sources that triggered their awareness towards the presence of DTCPS. It demonstrates a need to raise publics’ awareness by doctors towards this service. Same results were presented among Jordanian drive-thru community pharmacy consumers [17].

Participants showed positive attitudes towards the need for this service, as most of them expressed their support to establish drive-thru service at community pharmacies during COVID-19 time or later 44.8% (n = 253) and all over Malaysia 49.0% (n = 277) and believed that this will benefit all population 75.8% (n = 428). Same results were revealed in Saudi Arabia as customers of community pharmacies believed that drive-thru service will limit the risk of the COVID-19 pandemic and will be helpful for all population [24]. This contradicts with the previously reported before the emergence of COVID-19, that drive-thru pharmacy services are helpful only for a specific group of the population including geriatrics, disabled, and ill people [17,18].

Participants of this study preferred to request an order at a community pharmacy using drive-thru services through a drive-thru window and to receive the counselling while using this service briefly through the drive-thru window as well. This could be justified by a previously reported study in Taiwan that the drive-thru area allowed minimal noise and distraction, and the quality of the service could be fruitful [18].

As for participants’ perceptions towards the image of pharmacists following the introduction of the drive-thru service, about half of the participants believed that community pharmacists will have a good balance between the health of patients and the business side of their work. Nonetheless, the results of this study were consistent with a previous study conducted in Jordan, where it was found that 20.9% of drive-thru pharmacy consumers believed that community pharmacists will have a good balance between the health of patients and the business side of their work [17]. This is not a surprising result since the pharmacy profession changed the focus of practice from selling products to targeting patients’ care, over the past four decades [30,31].

The participants of this study perceived some differences between drive-thru and in-store drug refills. About half (51.1%) of the participants believed that the prescription might be filled more quickly in the drive-thru compared to in-store pharmacy services. Same results were documented previously [17,18,24]. However, participants believed that pharmacists providing drive-thru services, are less available to answer their questions, provide less written information, and cannot explain important points about prescriptions compared to in-store pharmacy services. This finding is supported by previous studies that indicated patient–pharmacist interactions were provided better during in-store drug refill service [32], and drive-thru service might reduce the interaction time between pharmacists and customers [17,33], especially for customers with limited English language proficiency [33].

The study revealed that most of the participants had positive perceptions towards this service during COVID-19 time. Participants believed that this service has several advantages during COVID-19. They believed that this service is helpful during COVID-19 to promote social distancing and to reduce the spread of the COVID-19 virus. Only one study discussed this service during COVID-19 in Saudi Arabia [24]. Moreover, it was previously reported before the emergence of COVID-19 that drive-thru service at community setting will provide more privacy, and fewer parking and traffic violations [17]. All previous studies concurred with our findings that drive-thru service is an accessible and convenient service that helps in getting prescriptions without delay, and less waiting time [12,15,17,24].

This study showed some socio-demographic factors that are associated with the publics’ use of drive-thru services during COVID-19. People with age above 55 years were more likely to use this service, in contrast to Lee and Larson [32], where younger people were more likely to use drive-thru services. This could be attributed to the time of conducting this study before the emergence of COVID-19 by Lee and Larson. Males, being married, and having children were more likely to use the drive-thru services, similar findings were documented in Jordan [17]. In Jordan, generally adult males (either married or not take responsibility for the daily life needs of their families) and because of being so busy, they tend to search for the fastest and most time-saving services for their needs [17]. Additionally, parents having children prefer using services that ensure keeping their children with them without the need to leave them alone in cars [17]. Other explored socio-demographic factors need more studies for further assessment, as we found that having a bachelor’s degree, being employed, and not being from a medical team or student, positively affected the Malaysian publics’ use of the drive-thru pharmacy services during COVID-19.

This is the first study that assessed the socio-demographic factors associated with the participants’ perceptions towards DTCPS during COVID-19. Non-Malaysian nationality and age above 55 years negatively affected participants’ perceptions towards DTCPS, further studies are needed in this regard.

There is a shortage of prior research studies about the use of DTCPS and its disadvantages [17,34]. The participants of this study believed that using this service may contribute to some disadvantages and mostly restricting the opportunity to interact with the pharmacist, and difficulty in providing drug information/counselling to patients (especially written information), same findings were reported by previous studies [15,17,32–34]. Furthermore, the participants believed that drive-thru community pharmacy services are mainly suitable for refilling prescriptions and for buying OTC products. This finding concurs with a study by Abu Hammour et al., where customers of community pharmacies believed that drive-thru pharmacy service is only suitable for purchasing OTC and refilling prescriptions [17]. This could be attributed to the believed disadvantages while using this service among the participants either in this study or previous studies [15,17,32,34], which are fewer interactions between pharmacists and patients and less provided written drug information to patients.

There were several limitations of our study. Firstly, to prevent the spread of COVID-19, the instrument was distributed online, and the participants filled it out via Google Forms, responses from the areas without the accessibility of the internet may not be captured. This may lead to demographic selection bias. Secondly, the instrument relied on participants’ self-rated assessment of their own perceptions, which may have resulted in an overestimation of the results. Furthermore, there is a lack of prior research studies on the DTCPS as previous studies only centered on Malaysian public hospitals such as Queen Elizabeth Hospital and Hospital Raja Perempuan Zainab II [14,15].

## Conclusion

This study showed positive awareness, attitudes, and perceptions towards DTCPS during COVID-19 in Malaysia among the general population, where they believed that those services were helpful during COVID-19 to enhance social distancing and to reduce the spread of the COVID-19 virus. However, concerns about poor communication between the pharmacist and the patient represented the main believed disadvantage of the use of DTCPS. This research provides insight into the impact of instilling drive-thru pharmacies in Malaysia including the accessibility, workflow, pharmacist-patient interaction, and the general image of pharmacists depicted by the public. Further studies are needed to assess the use of DTCPS during COVID-19 on a larger scale public and as well from the healthcare perspective. Furthermore, further studies on the full cost of DTCPS implementation are needed including the medicine preparation, manpower, and additional equipment to help the decision-makers in better assessment whether the DTCPS would make a worthwhile contribution to pharmaceutical care without having to expand the budget allocation.
